# Important tool in our rare disease toolbox: hybrid retrospective-prospective natural history studies serve well as external comparators for rare disease studies

**DOI:** 10.3389/fdsfr.2024.1418050

**Published:** 2024-12-18

**Authors:** Chinenye Ugoji, Julien Heidt, Joan Largent, Emily Bratton, Laura Hester, Sareh Keshavarzi, Stuart Turner, Christina Mack

**Affiliations:** ^1^ IQVIA, Epidemiology, Durham, NC, United States; ^2^ The Janssen Pharmaceutical Companies, Titusville, NJ, United States; ^3^ IQVIA, Biostatistics, Montreal, ON, Canada; ^4^ Rocket Pharmaceuticals Inc., Cranbury, NJ, United States

**Keywords:** rare disease, natural history studies, real-world data, hybrid design, external comparators, clinical development

## Abstract

Natural history studies (NHS) can support regulatory decision-making at different stages of the drug product life cycle and are especially important in the context of rare diseases, which are associated with not only delayed or erroneous diagnoses but also a lack of approved treatments. Real-world evidence can fill knowledge gaps and support treatment decision-making, thereby benefiting affected patients. In this context, there are three important options for NHS design: retrospective, prospective, and cross-sectional. Each of these has been successfully used to support regulatory approval as external comparator arms (ECAs) for clinical studies, especially single-arm trials (SATs). While longitudinal data obtained from retrospective or prospective designs have been more commonly used and have been the focus of regulatory guidance documents, hybrid designs that combine retrospective and prospective data collection are particularly powerful for rare disease studies. This is due, in part, to the smaller number of patients impacted by each rare disease. In these settings, retrospective or prospective data collection alone may not be sufficient or fit-for-purpose for an external comparator. Rather, a strategic combination of all available data, regardless of timing, can deliver the right information of the desired quality and completeness to answer these important questions and support regulatory evidentiary needs. For instance, patients included in retrospective studies may differ from recently treated patients in terms of disease severity, disease variants, clinical management, or other important aspects of the disease that may impact patient outcomes. Further, retrospectively collected data may lack specific data elements required to achieve adequate comparison with the treated group in single-arm studies. In the context of prospective designs, the recruitment of sufficient new patients for prospective follow-up may not be feasible or may be prolonged due to the rarity of the disease. Further, the potential for premature truncation of patient follow-up may result in insufficient longitudinal data, or prospectively collected data alone may not provide insights into the disease course for specific groups of patients. In these situations, primary data collection in a prospective study may be supplemented with retrospectively collected data from chart reviews, registries, or electronic medical record databases, either for the same patients, in an ambispective design, or for a different set of patients. These hybrid designs allow for broader and more robust contextual information on the patient journey and the natural course of the disease to be obtained, which can improve the suitability of the data as an external comparator for SATs or studies that lack internal control in situations where a prospective design alone might not be sufficient. Because retrospective and prospective data, or any two data sources that are being combined, may differ in availability and quality, there are unique challenges alongside the strengths of these designs. In this paper, we discuss considerations for the design, analysis, and conduct of hybrid NHS intended as ECAs for single-arm studies in clinical development programs for rare diseases.

## 1 Introduction

The recent publication of real-world evidence (RWE) guidance documents by the United States (U.S.) Food and Drug Administration (FDA) and the European Medicines Agency (EMA) reinforces the value of real-world data (RWD) for regulatory decision-making (U.S. Food and Drug Administration 2023a; U.S. Food and Drug Administration 2020a; U.S. Food and Drug Administration 2023b; U.S. Food and Drug Administration 2023c, European Medicines Agency, 2020a; European Medicines Agency, 2023b). Regulatory authorities have endorsed the use of external comparator arms (ECAs) to demonstrate effectiveness in certain circumstances. This approach has led to new drug approvals (U.S. Food and Drug Administration 2023b; Gray et al., 2020). To quantitatively assess a drug’s effectiveness, FDA regulations consider controls from “adequately documented” natural history studies (NHS) in comparable populations as valid for providing contextualization in ECA studies (21 CFR 341.126), and, in some cases, RWD from NHS can be used to provide confirmatory evidence to substantiate the results of a single adequate, well-controlled investigation (U.S. Food and Drug Administration 2023b). An ECA study involves the comparison of outcomes in participants receiving a test treatment according to a protocol to outcomes in a group of people outside of the trial who had not received the same treatment. An ECA can be a group of people, treated or untreated, from an earlier time (historical control) or during the same time period (concurrent control) but in a different setting (U.S. Food and Drug Administration 2023a). Although the guidance uses the term “concurrent control” in the context of both ECA studies and randomized internally controlled trials (U.S. Food and Drug Administration, International Council on Harmonization 2020b; U.S. Food and Drug Administration 2023a), in this paper, we distinguish them by using the term “concurrent comparator” for ECA studies. Historical (or non-concurrent) and concurrent comparators differ in the timing of the cohort assembly and data collection in relation to the initiation of the single-arm clinical study.

A natural history study is an observational study that is designed to follow the natural course of a disease and may include patients receiving the current standard of care (U.S. Food and Drug Administration 2021). Natural history studies have been used as historical and concurrent external comparators to demonstrate treatment effectiveness in drug development programs. In the context of rare diseases or rare subtypes of relatively common diseases (Arone, 2019) with no approved therapies, RWE generated from several types of natural history study designs has been used to support regulatory decision-making at different stages of the drug product lifecycle (U.S. Food and Drug Administration 2015; U.S. Food and Drug Administration 2021; European Medicines Agency, 2022; European Medicines Agency, 2020b; Finkel et al., 2014; Kolb et al., 2016; Kolb et al., 2017). Retrospectively collected natural history data from chart reviews, registries, or electronic medical records (EMR) and data from prospectively designed NHS conducted prior to the single-arm clinical study of a test treatment, intended for use as an external comparator, are considered historical or non-concurrent external comparators. Further, data from prospectively designed NHS conducted at the same time as the single-arm clinical study, intended for use as an external comparator, are considered concurrent or contemporary external comparators (Khachatryan et al., 2023; Burcu et al., 2020; Mack et al., 2020; Seeger et al., 2020; Rippin et al., 2024).

Longitudinal natural history data obtained from both retrospective-only and prospective-only designs have their own merits for use as external comparators and have been the focus of regulatory guidance documents; however, this delineation does not address situations in which retrospective or prospective data collection alone is either not sufficient or fit-for-purpose to serve as a valid external comparator for an ECA study. This scenario is particularly applicable to drug development for rare diseases, in which the rarity and severity of the disease, unmet needs, and/or practical/ethical considerations for conducting a placebo-controlled trial pose unique challenges to the design and conduct of NHS. Thus, understanding the natural history of rare diseases and generating suitable external comparators to support clinical development efforts may necessitate non-traditional, innovative approaches. A hybrid natural history study combines retrospective and prospective data collection, drawing on the strengths of both studies, while mitigating their individual limitations. While less common, these hybrid designs allow for broader and more robust contextual information on the patient journey and the natural course of the disease to be obtained in specific situations, improving the suitability of the data as an external comparator for single-arm trials (SATs) or studies that lack an internal control.

Given differences in retrospective and prospective data availability and quality, the combination of these data in hybrid studies presents unique methodological and practical challenges. The literature on hybrid NHS for regulatory decision-making is limited, and guidance on best practices for the design, conduct, and operational application of these studies as external comparators is lacking. In this paper, we discuss hybrid natural history study designs and the utility and key considerations for the planning, design, analysis, and conduct of hybrid studies intended for use as external comparators for single-arm studies in clinical development programs for rare diseases.

## 2 Case for hybrid designs

The goal of ECA studies is to compare subjects receiving an intervention within a clinical study with a group of patients external to the clinical study. This comparison allows discrimination between outcomes caused by the test drug and those caused by other factors unrelated to the test drug. A drawback to external comparators for demonstrating drug effectiveness is systematic differences between the external comparator and single-arm trial populations, including differences in data quality or bias due to differences in data collection (Jaksa et al., 2022; Seeger et al., 2020). Key variables that are needed to apply eligibility criteria may need to be “created equal” if different data sources are being used; this includes key prognostic factors, follow-up, management, measurement of key biomarkers, and an assessment of outcomes of interest. Longitudinal data obtained from both retrospective-only and prospective-only designs have been used for ECA approaches (Sola-Morales et al., 2023). While control groups of randomized trials are required to meet stringent eligibility criteria, the eligibility criteria for real-world studies are not as strict and are generally more relaxed in retrospective studies compared to prospectively designed studies. It is well known that less stringently assembled cohorts may be less comparable to the trial cohort and, thus, may lead to the observation of worse outcomes for patients in that cohort due to selection bias (U.S. Food and Drug Administration, International Council on Harmonization 2020b). Untreated patients in historical comparator groups (e.g., patients in a non-concurrent retrospective external comparator cohort) have also demonstrated worse outcomes compared to prospectively followed patients in the untreated arm or control group in randomized trials (Papageorgiou et al., 2017; Sacks et al., 1982; U.S. Food and Drug Administration, International Council on Harmonization 2020b), which may overestimate the effectiveness and safety profile of the test drug. Recognizing these limitations, the International Council for Harmonization of Technical Requirements for Pharmaceuticals for Human Use (ICH) guidance for industry, E10 Choice of Control Group and Related Issues in Clinical Trials, “supports the use of multiple external controls in situations where “no obvious single optimal external control exists … providing that the analytic plan specifies conservatively how each will be used in drawing inferences” (U.S. Food and Drug Administration, International Council on Harmonization 2020b).

Retrospective studies are typically explored as the first source of natural history data because the data already exist and, thus, the studies are relatively quicker and require less logistics compared to prospective designs. In rare disease, retrospective data may be the only viable means of accruing a large enough sample size to sufficiently evaluate trends and outcomes. Nonetheless, the utility of retrospective studies as external comparators may be limited by missing, incomplete, or inconsistent data; temporal changes in medical terminology or documentation practices; and changes in the characteristics of the patient population or disease (Nazha et al., 2021). Retrospectively collected data may lack specific data elements, including confirmatory diagnostic criteria and prognostic factors, required to achieve adequate comparison with the treated group in single-arm studies. Retrospective studies conducted prior to SATs may include patients who differ from more recently treated patients in disease severity, disease variants, the mode of diagnosis, diagnostic criteria, the natural course of the disease, or outcome assessment methods that may impact comparability to the treated group and accurate quantification of the study endpoints. For instance, the retrospective study of a genetic disease in which a variant was historically considered a variant of unknown significance (VUS) but later classified as a pathogenic variant may exclude VUS patients who, based on reclassification, need to be included in the single-arm trial.

Prospective studies can mitigate the limitations of retrospective studies. FDA guidelines for rare disease development programs emphasize “the need for prospectively designed, protocol-driven NHS initiated in the earliest drug development planning stages” (U.S. Food and Drug Administration 2023c) to allow for a standardized approach for data-monitoring, scheduled data collection frequency, and a standardized approach for clinical outcome assessments. In the context of rare diseases, however, the recruitment of sufficient new patients for prospective follow-up may not be feasible or may be prolonged due to the rarity of the disease, which may, in turn, delay the provision of timely advice needed for planning and implementing clinical trials of the investigational drug. Also, prospectively collected data alone may not provide insight into the disease course for specific groups of patients. Further, the potential for premature truncation of patient follow-up in prospective studies, either due to intercurrent events, the initiation of a clinical trial, or the availability of a potential treatment, may result in insufficient longitudinal data. In such situations, prospective data alone may not be sufficient to satisfy the objective of creating a suitable external comparator population for a single-arm trial. The strengths and weaknesses of prospective and retrospective NHS as external comparators are summarized in Table 1.

**TABLE 1:** Strengths and weaknesses of prospective and retrospective natural history studies.

Study design	Strengths	Weaknesses
Prospective external comparator	• Ability to tailor and/or augment capture of key study variables • Opportunities to mitigate missing, incomplete, or inconsistent data	• Recruitment of sufficient patients may not be feasible or may be prolonged in rare disease • May not provide insight into the disease course for specific groups of patients • Potential for premature truncation of patient follow-up either due to intercurrent events, initiation of a clinical trial, or availability of a potential treatment
Retrospective external comparator	• Relatively quicker time to data collection/extraction • Operationally more straightforward • Potential to accrue larger sample size	• Specific data elements required to achieve adequate comparison with the treated group in single-arm studies may not be available • More likely to have missing, incomplete, or inconsistent data • Temporal changes in medical terminology, diagnostic criteria, or documentation practices may hamper comparability with a more recent single-arm study • Evolution of disease characteristics, such as severity, fatality, and variants, may impact comparability with a more recent single-arm study

Because hybrid natural history study designs combine retrospective and prospective data collection, they can be considered for specific situations in which retrospective or prospective data alone are not sufficient for use as an external comparator. In these situations, primary data collection in a prospective study may be supplemented with retrospectively collected data from chart reviews, registries, or EMR databases, either for the same patients (in an ambispective design) or for a different set of patients.

A recent example of a hybrid natural history study that has been successfully used to obtain regulatory approval for a rare disease indication comes from Nulibry^®^ (fosdenopterin), approved by the FDA and the EMA (U.S. Food and Drug Administration 2021; European Medicines Agency, 2022) for the treatment of molybdenum cofactor deficiency (MoCD) Type A, an exceedingly rare, fatal, autosomal recessive disease with an estimated U.S. prevalence of approximately 50 patients, all under 10 years of age. The natural history study combined retrospective and prospective data on untreated patients with MoCD Type A (Spiegel et al., 2022). A total of 37 untreated patients with confirmed MoCD Type A—20 (54%) deceased patients in a retrospective cohort and 17 (46%) living patients (of whom 14 enrolled in a 12-month prospective study) were included in the natural history study. For the external comparator analysis, 18 patients in the hybrid natural history cohort were genotype-matched to 13 treated patients in the Phase II trial, and a relatively large treatment effect size was demonstrated for the reliable and objective endpoint of mortality (overall survival). A 3-year estimated survival probability of 53% [95% confidence interval (CI), 28%–73%] was noted in the untreated genotype-matched analysis set *versus* 84% (95% CI, 47%–96%) among fosdenopterin-treated patients (U.S. Food and Drug Administration 2021). The FDA concluded that this comparison of treated and untreated patients constituted an adequate, well-controlled investigation (U.S. Food and Drug Administration 2021). The EMA noted there was potential for selection bias in the retrospectively collected data, but ultimately considered it unlikely that selection bias had occurred (European Medicines Agency, 2022).

## 3 Types of hybrid designs

**FIGURE 1:**
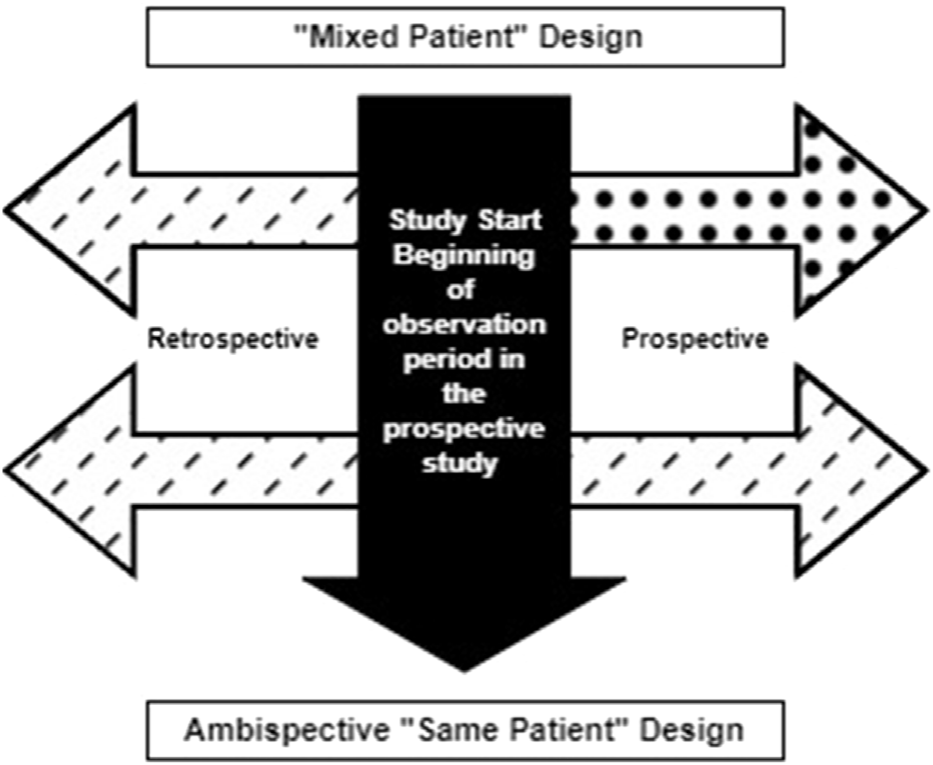
Variations in hybrid natural history study designs.

Conceptually, hybrid NHS can be broadly classified into two categories: a hybrid “mixed patient” design, in which retrospective and prospective data are collected on different sets of patients, and a hybrid “same patient” or ambispective design, in which prospective and retrospective data are collected on the same set of patients (Figure 1). These designs may also be combined in a single study, as needed. We will briefly outline each of these designs and discuss their applicability, strengths, and limitations. Then, we will outline a few of the study designs and analytic considerations applicable to the conduct of “same patient” hybrid designs, as these are quite novel and as existing literature on these designs is limited.

### 3.1 “Mixed patient” design

In the “mixed patient” design, the external comparator population includes a set of patients followed prospectively either in real-world settings or in a clinical trial, and another set of patients for whom retrospective longitudinal data are available. This design, in theory, allows for an increased sample size and quicker patient accrual compared to a standalone prospective or retrospective study of the same disease. For specific situations in which patient demographics or the disease variant is known or thought to have evolved over time, the hybrid “mixed patient” design may allow for more diversity in the patient population and representation of different disease strains, variants, subtypes, or severity in the external comparator population. This would be particularly relevant for clinical development programs that target various phenotypes of a disease. Furthermore, insights derived from readily available retrospective data, such as patterns of routine clinical follow-up and management of patients, the frequency of laboratory assessments and other investigations, types, and frequency of clinical outcome assessments (CoAs), may inform the design of the prospective follow-up period and the single-arm trial (e.g., relaxing allowable windows for assessments, where no standard of care has been previously established). Careful consideration should be given to how care patterns in the retrospective data inform prospective design so as not to introduce further bias. While heterogeneity in patient demographics and disease variants is advantageous, the combination of patients in retrospective and prospective studies for use as an external comparator would require reasonable congruence in the frequency of assessment, as well as disease diagnostic and outcome assessment methods. As with all NHS intended to serve as external comparators for single-arm clinical studies, a patient’s time of study entry or enrollment in the natural history study may differ from the definition of time zero or the index date (hereafter referred to as time zero) in the ECA study. Section 5.2 describes the considerations for the definition of time zero for the ECA. Given that the retrospective and prospective cohorts include unique patients, the eligibility criteria and definitions of study entry into the natural history study should be similar for patients in both cohorts. Due to temporal differences in time zero for patients in both cohorts, combining the data as an external comparator would require that key eligibility criteria and prognostic factors are available and applied comparably in both populations. For instance, if genetic test results obtained using a specific method were required as an inclusion criterion for the single-arm trial, genetic test results using the same method or one that is comparable to the method used in the single-arm trial should be available in the retrospective and prospective cohorts.

### 3.2 Ambispective “same patient” design

The ambispective “same patient” design involves the collection of data prospectively and retrospectively for the same set of patients. While many studies use this approach (e.g., look-back period to assess medical history, comorbidities, diagnosis journey), in this instance, we are considering the ambispective design for an ECA study, such that the RWE will be used in comparison to a treated population. In this use case, data availability and methods to control for bias become paramount.

**FIGURE 2:**
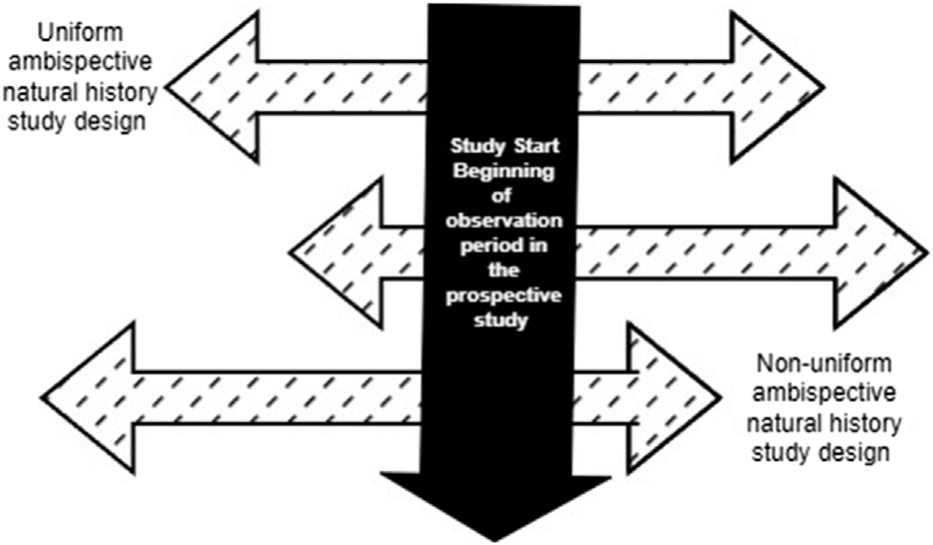
Variations of Retrospective and Prospective follow-up in an ambispective “same patient” natural history study design.

In an ambispective design, the duration of the retrospective and prospective data collection may be uniform or vary based on the study needs, as well as ethical and practical considerations**.** In a uniform “same patient” hybrid design, the same duration of retrospective and prospective data is collected for each patient. For example, a study that requires 24 months of longitudinal data for the external comparator could pre-specify that data from 12 months of prospective patient follow-up be combined with 12 months of retrospective data (e.g., from electronic health record data) on the same patient prior to enrollment or the beginning of the observation period in the prospective study. Conversely, in a non-uniform ambispective design, the duration of the prospective and retrospective follow-up may vary for each patient (Figure 2). In this scenario, a study that requires 24 months of longitudinal data may include patients with varying combinations of retrospective and prospective follow-up (e.g., 6 + 18, 10 + 14, 12 + 12, 20 + 4) to obtain the 24-month longitudinal data required. The beginning of the observation period for a patient in this ambispective “same patient” natural history study design will be the beginning of the retrospective follow-up period, as defined by the specific study based on clinical and practical considerations. However, this may be different from time zero for the ECA study, which corresponds to when the patient meets the eligibility criteria for inclusion in the ECA study. In rare diseases with very low prevalence and incidence, this design can allow researchers to increase the available assessment window by supplementing insufficient prospective data with retrospective data. Compared to a prospective-only design, this design can significantly reduce the time frame for the study by increasing the person-months of follow-up. This approach would be particularly relevant for clinical development programs in which it may not be feasible to obtain sufficient and/or relevant prospective longitudinal data on patients. A few scenarios include anticipated truncation of patient follow-up due to the availability of a potentially beneficial clinical trial, loss to follow-up or death, or the need to observe a specific interval defined by age or calendar time in patients diagnosed with the disease. For instance, a clinical development program interested in observing the natural history of disease in children in their first 2 years of life may enroll children aged 24 months of age or less in an ambispective hybrid natural history study. An eight-month-old infant enrolled in this ambispective study design will have 8 months of retrospective data and an additional 16 months of prospectively collected data, while a 12-month-old enrollee will have 12 months of retrospective and 12 months of prospectively collected data. As noted in the mixed patient design, study entry in the natural history cohort at birth may differ from time zero in the ECA study if the eligibility for the drug trial differs from the natural history study entry criteria. In both uniform and non-uniform ambispective designs, the time zero for each patient in the ECA will depend on the time point at which the patient meets the trial eligibility criteria. If, for instance, patients must be at least 6 months old to receive the trial drug, time zero for untreated patients in the ECA will be defined by the age at treatment initiation when patients in the retrospective or prospective cohorts received the trial drug. Notably, in clinical development programs for rare diseases with no widespread screening tests and fewer discrete timepoints for disease onset, the timing of study entry/enrollment into the natural history study and the definition of time zero for the ECA study may be more nuanced. In these scenarios, patients may only be screened and diagnosed when they manifest clinical symptoms of the disease, which vary by patient.

As with the “mixed patient” design, the combination of prospective and retrospective data in the ambispective “same patient” design would require that data on prognostic factors and outcomes be available and measured comparably in the retrospective and prospective follow-up periods. To build the external comparator cohort for the single-arm trial, a patient may meet the eligibility criteria within one or more windows during follow-up. Thus, investigators may choose a time zero within the eligibility window that provides the most complete data required for the ECA study. Inconsistencies in time zero selection and the application of eligibility criteria may introduce selection and misclassification bias, particularly if investigators inadvertently select slices of retrospective data that result in the untreated cohort looking artificially worse than the treated cohort. Additional examples of such potential biases in this design are provided in the next section.

### 3.3 Combination of “mixed patient” and ambispective “same patient” design

**FIGURE 3:**
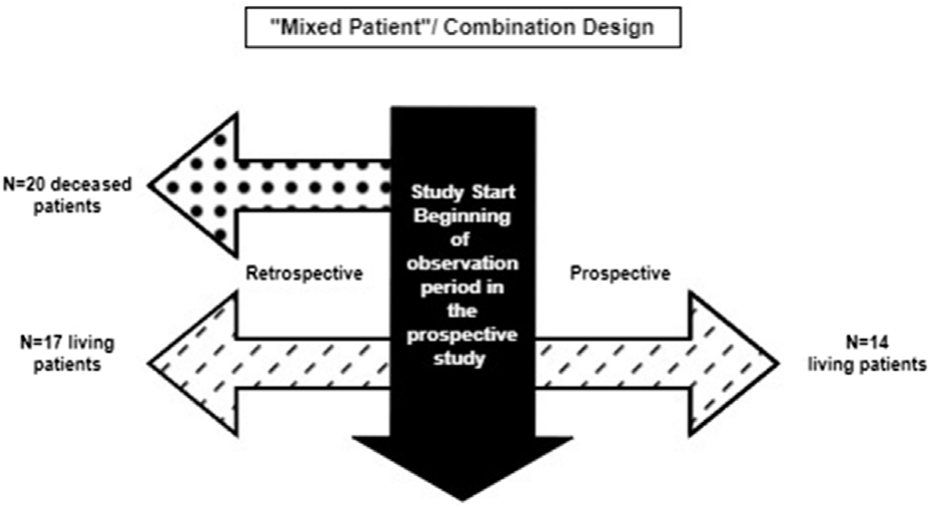
Combination of retrospective only and ambispective data collection in the Nulibry^®^ hybrid natural history study design.

This design combines the two aforementioned approaches and may include a few patients followed prospectively only, some with retrospective data only, and a few who have both retrospective and prospective follow-up data. The hybrid natural history study that supported the ECA study and regulatory approval for Nulibry (U.S. Food and Drug Administration 2021; European Medicines Agency, 2022) is an example of a hybrid “mixed patient” design. This design may be applicable to clinical development programs for rare diseases for which retrospective data, even for deceased patients, are informative and can be augmented with additional prospective data on new and pre-existing patients. This approach maximizes the data available for providing contextual information beyond the hybrid “mixed patient” and ambispective “same patient” designs. The natural history study of sulfite intoxication disorders due to molybdenum cofactor deficiency that was used to support Nulibry approval employed a “mixed patient” design combining retrospective data for 37 patients (17 living, 20 deceased) and prospective data for 14 of those 17 (Figure 3); 18 of the 37 were genotype-matched to treated patients in the external cohort. This combination design allowed observation of the first year of life for all 37 patients, including those who did not survive to Year 1, possibly representing a unique disease phenotype or severity (Spiegel et al., 2022, U.S. Food and Drug Administration 2021). Table 2 provides examples of the applicability of specific variations of hybrid natural history designs to different scenarios.

**TABLE 2:** Potential applicability of specific variations of hybrid natural history designs.

Study scenario	Type of hybrid natural history study		
	“Mixed patient”	Ambispective “Same patient”	Combination
Disease- or condition-specific considerations			
Unmet treatment need, very rare disease	X	X	X
Disease of interest is rapidly fatal, potential limiting the duration of prospective follow-up		X	X
Disease has variants that have evolved over time, but the investigational drug is intended to target multiple variants	X		X
Ethical or practical considerations			
Truncation of prospective follow-up is anticipated, e.g., due to timing of new clinical trial that may enroll patients in the NHS		X	X
It is ethical/feasible to enroll a few patients in a placebo/standard of care arm under the trial protocol, but the enrollment rate is expected to be low	X		X
Previous retrospective studies were informative but had a limited sample size or did not employ the same diagnostic methods as single-arm trials	X	X	X

## 4 Considerations for design of hybrid natural history studies

### 4.1 Feasibility

#### 4.1.1 Regulatory feasibility

Given that ECA studies are reserved for special situations in which a randomized controlled trial (RCT) is not ethical or feasible, considering the regulatory feasibility of using a hybrid natural history study cohort as an external comparator is imperative. Regulatory feasibility is the process of assessing the ability of RWD to meet regulatory effectiveness evidentiary standards by evaluating the characteristics of the clinical development program (Campbell et al., 2023). As there are unique operational and methodological challenges associated with these hybrid designs, it is important to assess first whether an external comparator approach is appropriate and then if prospective data alone or retrospective data alone would be fit to meet regulatory objectives.

Regulatory feasibility may leverage a targeted literature review to garner insights into how potential analogs or other rare disease therapies have used hybrid designs as evidence in a submission package for marketing authorization. In lieu of strong precedent, it is also recommended that sponsors stay abreast of evolving regulatory guidance to identify necessary components of the regulatory rationale and key criteria necessary to meet regulatory objectives and inform early discussions with regulatory review divisions during the study design phase. For hybrid studies in particular, a gap analysis of the proposed evidence strategy is critical. Special consideration should be given to changes in the standard of care over time for a given disease, such as changes in the diagnostic criteria, treatment patterns, or outcome assessment. A significant evolution in the standard of care over time, for example, may imply historical retrospective data alone are not sufficient; however, this should be considered in the context of other practical considerations, such as the recruitment of sufficient new patients with sufficient follow-up. If gaps in evidence (e.g., patient population, treatment history) still exist, there may be a regulatory rationale for the hybrid study design approach that can be further investigated through a feasibility assessment of putative data sources and subsequently discussed with regulators as part of a robust early engagement strategy.

#### 4.1.2 Data source feasibility

Data feasibility narrows down the universe of potential data sources and provides transparency to regulators around why data sources were included or excluded. Feasibility should be driven by an understanding of the causal structure of the research question, including operational definitions and the specification of minimum criteria (Gatto et al., 2019). The process of determining the feasibility of data for a hybrid natural history study may begin with identifying a long list of potential data sources for a given indication. This may be done through a data landscaping exercise or some other systematic approach to identifying potential data sources. Depending on the planned approach, retrospective and prospective data may come from the same or different sites/data sources. Identified data sources may be based on the planned single-arm trial sites, either at the same sites or different sites in similar geographic locations (also considering the type of site, e.g., academic *versus* community hospital). Rare disease studies typically require pooling data from multiple data sources to bolster the sample size and/or reflect the trial geographic coverage more closely, and hybrid studies have the additional nuance of pooling retrospective and prospective data collected at different time periods from the same or different sources. These data sources will have varying degrees of data quality and management that must be considered. Thus, it is important to not only independently assess the quality of each data source across multiple dimensions but also do so in the context of the other data sources to understand potential limitations or barriers to data aggregation that may exist, in line with established guidelines for the multidimensional evaluation of data quality (Section 6.1) (Castellanos et al., 2024; Lerro et al., 2024; European Medicines Agency, 2023a).

Next, a focused assessment is carried out on a select set of promising data sources. The criteria for selecting data sources to be contacted for data feasibility should be specified clearly and transparently. The rationale for this process is grounded in the guidance; in particular, sponsors should describe in the study protocol, or as an appendix to the protocol, the data sources evaluated when designing the study, including results from feasibility evaluations or exploratory analyses of those data sources. Investigators should also provide a justification for selecting or excluding relevant data sources from the study and describe how the choice of the final data sources, study design elements, and analytic approaches aligns with the research question of interest and demonstrate that these were not selected to favor particular study findings (U.S. Food and Drug Administration 2023a). Documenting this rigorous process and detailing potential limitations of the data sources provides the justification and transparency that are called for in the guidance.

A robust data feasibility assessment early in ECA study planning can mitigate key risks to a real-world program. A key risk, particularly in the context of rare diseases, is an insufficient number of representative patients with adequate data quality for inclusion in analyses, including pre-specified and powered subgroup analyses. Through feasibility assessment, one can estimate the number and quality of patients likely to be eligible and the impact of potential changes on the standard of care regarding patient counts. This often includes looking at the distribution of patients across ranges of assumptions to help inform certain decisions about eligibility criteria, illustrating how feasibility and study design can inform each other iteratively. To that end, an investigator may initiate feasibility with the intention of conducting a retrospective-only or prospective-only study and determine, based on feasibility results, that a hybrid approach is better suited to meet regulatory objectives.

#### 4.1.3 Study design feasibility

Data source selection and study planning for NHS intended to serve as external comparators should ideally align with preclinical/clinical development and should aim to reflect the planned drug-intervention clinical trial in terms of planned study-related assessments. Per guidance from the FDA, “sponsors should finalize a study protocol before initiating the ECA study, including selection of the external control arm and analytic approach, rather than selecting an external control arm after the completion of a single-arm trial” (U.S. Food and Drug Administration 2023a). This recommendation from the agency stems from the need to bolster comparability between the trial and external comparator designs, promoting a parallel and integrated design process rather than the conceptualization of an external comparator cohort following the finalization/initiation of a single-arm trial, which may further contribute to issues with cohort comparability and exchangeability from the design perspective. This concern regarding comparability between cohorts is a fundamental consideration for investigators that is applicable to both retrospective and prospective designs and is similarly applicable within the external comparator cohort itself when combining prospective and retrospective RWD collection.

Regulatory, data, and study design feasibility will ultimately inform regulator interaction. Discussions with regulators should aim to provide a rationale for the use of a hybrid design and discuss the choice of a hybrid design (e.g., same vs mixed patient). A detailed assessment of fit-for-purpose for both prospective and retrospective data sources should be provided, acknowledging how differences in data reliability (inclusive of data accuracy, completeness, provenance, and timeliness) informed data source selection and key study design decisions. Many decisions regarding the hybrid study design will be context specific and depend on the indication in question, making early and continued regulatory engagement essential to de-risking the external comparator program and informing a robust regulatory strategy. The issues and challenges associated with hybrid study designs will be discussed in the next section.

## 5 Study design considerations

The study design phase presents the best opportunity to anticipate and reduce the risk of bias in the study. In the design phase for hybrid studies that are intended to be used as external comparators, the study design may be driven by current knowledge about the disease, preliminary data, and identified gaps from previous studies, as well as the overarching goals and objectives of the ECA study. In this phase, investigators will need to identify feasible data sources and/or sites for retrospective and prospective data collection, specify eligibility criteria for the external comparator cohort, and clearly define all exposures and outcomes/endpoints of interest. Further, investigators should have clarity on the availability of key prognostic variables and variables needed to address confounding in the ECA study, consider which hybrid design is most appropriate for the ECA, and how to best utilize the retrospectively and prospectively collected data. The challenges that are specific to hybrid designs, including potential biases, should be anticipated at this stage, and mitigation approaches to address them should be woven into the study protocol.

### 5.1 Understanding study population

To minimize bias, external comparator cohorts are expected to be comparable to the treatment arm of the single-arm trial across baseline, demographic, and prognostic factors that may influence the study outcomes. To ensure comparability, matching, weighting, or stratification techniques using key prognostic factors are often employed; however, there are specific considerations for the hybrid design use case. In hybrid study designs, in addition to ensuring the comparability of the hybrid external comparator cohort to the treatment arm of the single-arm trial, the comparability of the retrospective and prospective study populations on the availability and measurement of key prognostic factors is important and should be considered during the design phase. Notably, the heterogeneity of patient populations in the retrospective and prospective study populations in mixed patient designs can be an advantage in specific situations, such as when hybrid studies allow the inclusion of subgroups of patients or disease variants that may be missed in prospective-only or retrospective-only designs. However, the key attributes with prognostic importance, such as eligibility and/or diagnostic criteria, as well as measurement of biomarkers and study outcomes, should ideally be similar during the retrospective and prospective periods of both hybrid “mixed patient” and ambispective “same patient” designs. Given that a perfect alignment of these attributes in retrospective and prospective data is rare, investigators may minimize potential bias due to these differences by pre-specifying critical data, including key prognostic factors in the protocol that will be used for matching or weighting the datasets in the ECA study, and ensuring that these variables are available in the retrospective and prospective data. Fragmentation of patient health information due to the receipt of care from multiple providers and sometimes across multiple health systems and settings that were not intended for research purposes is a challenge in real-world studies. Where practical, ethical, and feasible, missing key prognostic variables may be obtained by linkage to external RWD sources or tokenization of patient data (Dagenais et al., 2022) and should be pre-specified in the study protocol. Lastly, if the external comparator cohort is indexed on a comparator treatment for the trial (e.g., standard of care), temporal and operational differences in treatment decisions, treatment patterns, treatment interruption, patient care, and monitoring in the retrospective and prospective follow-up periods may have prognostic importance and should also be considered.

### 5.2 Selection of appropriate time zero

Time zero is the beginning of the observation period for assessing study endpoints, including intercurrent events in the ECA study. As discussed previously, this date may be different from the study entry or enrollment date for patients in the natural history study cohort (Section 3.1). This is because, while the natural history study seeks to understand the complete spectrum of the disease from the earliest diagnosis or symptomatic manifestation of the disease, the ECA study is based on the ideal time at which a therapeutic intervention may be feasible or most beneficial to the diseased population. In RCTs, randomization prior to assignment to the treated or untreated groups ensures that time zero is meaningful and comparable across treatment arms. In ECA studies, the absence of randomization makes the selection of a time zero that is comparable to the start of treatment in the single-arm trial challenging (Hatswell et al., 2022; Rippin et al., 2022). In some cases, patients may be qualified for inclusion in the ECA at multiple time points (e.g., due to multiple lines of therapy in cancer treatment), adding further complexity. Incomparability in time zero selection in the external comparator and treated populations of ECA studies, particularly for studies in which treatment is not immediately preceded by a discrete event, can result in bias due to immortal time (U.S. Food and Drug Administration 2023a; Suissa, 2008; Lambert et al., 2023).

In the context of hybrid studies with combined data, the selection of time zero is even more nuanced, as potential differences in diagnostic methods, monitoring, management, and follow-up of patients in the retrospective *versus* prospective phases of the study introduce additional complexities. For instance, consider an ECA study of a novel therapy intended to prolong survival among patients affected by a rare and fatal genetic disease with no available newborn screening. Patients with the disease are typically diagnosed during childhood or early adulthood, when symptoms become apparent, and are managed symptomatically. In the single-arm trial, subjects who meet the eligibility criteria, including confirmed diagnosis by genetic testing and non-response to symptomatic management, are identified, enrolled, and treated with the test drug, and time zero for the ECA study is defined as time since non-response to symptomatic management. The hybrid external comparator cohort identifies and follows up on patients who were historically diagnosed and managed in real-world settings (ambispective “same patient” design) and may include newly diagnosed patients who are eligible for prospective follow-up (hybrid “mixed patient” design). In this scenario, bias due to immortal time may result if there is a time window between the establishment of non-response to the symptomatic treatment and the receipt of the investigational drug. Although this bias is not unique to hybrid designs, differences in the definition criteria for non-response in the retrospective and prospective arms of the hybrid design *versus* the single-arm trial may make establishing time zero even more challenging. Differences in the availability and timing of genetic testing in the retrospective patients/period, prospective patients/period, and trial population may also impact the determination of study eligibility and time zero. To mitigate the risk of biases, it is important that investigators pay attention to these considerations during the design phase of hybrid NHS.

As discussed previously, a potential use case for hybrid designs is a scenario in which premature truncation of follow-up in the prospective study due to an emerging clinical trial or another competing factor is anticipated. In this scenario, an ambispective “same patient” design may be appropriate, and time zero may be adjusted based on the required length of longitudinal data desired and how much prospective follow-up is feasible or accrued (Figure 2). Similarly, in scenarios in which potential differences in the quality and completeness of data in the retrospective and prospective segments of RWD intended for use as an external comparator are a concern, time zero may be altered in an ambispective “same patient” design to optimize data quality and completeness in the combined longitudinal follow-up data that will be used as an external comparator. Where patients meet the eligibility criteria within one or more windows during follow-up, investigators may choose a time zero within the eligibility window that provides the most complete data required for the ECA. In situations in which a patient has more than one eligible time zero option with similar levels of data completeness, an investigator will need to decide which option to use in the analysis. To avoid the potential for biases, it is important to pre-specify during the study design phase what algorithm(s) will be applied to time zero selection during the analysis. However, it is noteworthy that this approach may be more applicable to the ECA study of therapies for rare diseases for which disease onset is a non-discrete event and for which there are no available treatments; thus, time zero is not tied to a discrete event other than an arbitrary date of confirmed diagnosis of the disease. Use of this data optimization approach requires an abundance of caution to ensure that all study eligibility criteria are met at the time point of the adjusted time zero. For instance, if patients must be 18 years of age or older to be included in the study, the adjustment of time zero to optimize RWD use must ensure that patients are 18 years of age or older at the beginning of the observation period.

For “mixed patient” ambispective hybrid study designs that involve a combination of unique patients in the retrospective and prospective follow-up periods, time zero for the ECA study should be as similar as possible for patients in both the prospective and retrospective cohorts, as well as the single-arm clinical cohorts. For instance, a natural history study of a genetic disease for which newborn screening is available and that is typically diagnosed at birth may enroll and follow patients from birth for a pre-specified period. Patients in the prospective study will be followed from birth, and patients in the retrospective study will also enter the cohort at birth, regardless of their age at the time of cohort assembly. Time zero for the ECA will depend on the age of eligibility for the trial drug. If, for instance, patients must be at least 6 months old to receive the trial drug, time zero for untreated patients in the ECA will be defined by the age at treatment initiation if patients in the retrospective or prospective cohorts received the trial drug.

### 5.3 Ascertainment of outcomes and endpoints

In ECA studies, it is important to ensure that clinical and non-clinical study endpoints are measured comparably between the trial and external comparator populations. The same criteria for the evaluation and timing of outcome assessments should be applicable across both arms of the ECA study (U.S. Food and Drug Administration 2023a). In the hybrid NHS that will be used as external comparators, ensuring comparability between the assessment or measurement of endpoints in the retrospective and prospective NHS is critical. Hybrid designs should demonstrate internal consistency in outcome assessments by ensuring that the same criteria for evaluation and timing of outcome assessments are applicable in the retrospective and prospective segments of both hybrid “same patient” or “mixed patient” designs. This internal consistency in outcome assessments is an important criterion for combining prospective and retrospective data in hybrid NHS used as external comparators. For example, it may be possible to collect liver biopsies (i.e., a gold-standard endpoint) in the prospective study, but in the retrospective data, only liver scans or biomarkers are available. In such situations, a re-evaluation of the intended approach may be required. Similarly, if the endpoint is a change in biomarkers over time, differences in the frequency of visits during the prospective and retrospective periods may introduce differences in the intervals between biomarker measurements. It is important to consider differences in the window between measurements in the prospective *versus* retrospective periods of follow-up, as well as the impact of these differences in the estimation of the specific endpoint. Patient-reported outcomes are often an important outcome in NHS for rare diseases but are also less likely to be completely and consistently measured in real-world retrospective data sources. Lastly, there may be changes in the definitions of endpoints over time, which will impact interpretability/comparison between retrospective and prospective data. This is even more relevant in the context of rare disease, including rare disease oncology, where emerging knowledge and advancements in science lead to an evolution of endpoint definitions.

## 6 Analytical considerations

As with any real-world study, characterizing data quality and completeness is critical to assessing the fit-for-purpose of a given data set for regulatory decision-making. Unique to these hybrid studies is the combination of retrospective and prospective data and the associated inherent differences in the data’s characteristics. It is important that investigators pre-specify and implement quality control measures and data integration strategies that improve the validity and reliability of the retrospective and prospective data. These include validation checks, inter-rater reliability assessments, the standardization of variables across datasets, routine monitoring, and periodic data reviews to identify and mitigate errors. Analytical decisions will depend on anticipated gaps identified from data feasibility assessments, as well as unanticipated gaps identified during data collection. Statistical and analytic considerations for ECA studies, including power and sample size considerations and the application of causal inference methods, the handling of missing data, partial index dates, and unmeasured confounding, have been previously described (Rippin et al., 2022). The authors highlight three distinct features in ECA studies that warrant attention during the design and analytical stages: (1) missing data, including unmeasured confounding; (2) differential covariate and endpoint(s) measurement approach and timings; and (3) correlated data due to repeated patient eligibility for inclusion in the external comparator cohort. Missing data and unmeasured confounding impede the adjustment of confounding in ECA studies. In addition to these previously described considerations, the use of hybrid external comparator data in ECA studies requires a few additional analytical considerations that we discuss below.

### 6.1 Combining retrospective and prospective data in hybrid designs

During the design phase, investigators will need to determine how best to utilize the retrospectively and prospectively collected data based on the findings from the feasibility assessment. Where the data need to be combined, the head-to-head combinability of retrospective and prospective data in the “same patient” design or the combination of the retrospective and prospective cohorts in the “mixed patient” design must be carefully considered. Although no clear-cut criteria for data combinability have been developed in the context of hybrid designs, it is important that investigators who intend to use a hybrid external comparator for regulatory submission consider the heterogeneity of measurements and outcomes between the prospective and retrospective patient populations, as strong assumptions are needed to combine these patients into one comparator arm. Backenroth et al. describe a framework for pooling datasets into a real-world comparator cohort: pre-specification of research questions and pooling processes; assessment of data set eligibility (e.g., meta-data, variables of clinical relevance, non-outcome characteristics, sample size); outcome analysis, including assessment of heterogeneity in outcomes; and pre-specified sensitivity analyses. A fundamental assumption of these pooled analyses is that comparison of a single-arm trial to two different real-world comparator cohorts should yield statistically identical results (i.e., no heterogeneity in outcomes). Notably, patient heterogeneity is a desired attribute of hybrid external comparator designs. Thus, while testing for heterogeneity in outcomes between studies is a standard step in the pooling process and is important when creating a real-world comparator cohort from disparate datasets (Backenroth et al., 2023), an ideal framework for combining retrospective and prospective data in a hybrid external comparator may require relaxing the statistically identical results assumption.

### 6.2 Missing data or unmeasured confounding

ECA studies that intend to use hybrid data as an external comparator will need to consider the extent and differences in data missingness and unmeasured confounding during the retrospective and prospective periods (in the ambispective same patient designs) or patients (in the hybrid mixed patient design). The frequency of missing data (e.g., partial dates) is expected to be less in the prospective period of the study than in the retrospective period, given the ability to implement systematic follow-up and data-monitoring activities. When patterns of missingness are understood, multiple imputation (MI) may be applied to obtain the covariates needed to address confounding and adjust for bias. While MI across the external comparator and single-arm trial cohort has the advantage of utilizing information from the single-arm trial to inform imputation and can be applied to hybrid mixed patient designs, performing MI within the ambispective same patient external comparator cohort may be explored, where the information in the prospective data may be used to inform imputation of missing data during the retrospective period. Regardless of the MI approach selected, it is important that investigators discuss the appropriateness of the chosen approach with regulatory authorities during the development of the statistical analysis plan.

### 6.3 Sensitivity analysis

As with other studies using RWD, sensitivity analyses may be included to test the robustness of various assumptions made in the study design and analysis to the study results. For instance, sensitivity analysis may be used to determine whether missingness is informative and may inform MI techniques. In ambispective “same patient” designs, premature truncation of patient follow-up may necessitate a non-uniform combination of retrospective and prospective data to generate the required length of longitudinal follow-up. This approach results in an adjusted time zero that differs from the beginning of the observation period in the prospective study. To test the impact of alternate baseline assessment time points on study results additional sensitivity analyses may be used, such as excluding patients with retrospective data or utilizing only sites that are not participating in the trial if there are concerns about selection bias.

## 7 Conclusion and future directions

Hybrid NHS are a strategic external comparator approach that can support regulatory approval of drugs intended for rare diseases or rare subgroups of common diseases. This approach is a particularly important tool when prospective or retrospective data collection alone is not feasible, sufficient, or fit-for-purpose. Hybrid NHS provide extended options to investigators to leverage RWD as part of the totality of evidence.

The conduct of hybrid studies and their utility in ECA studies may be accompanied by additional operational and methodological challenges, beyond those of standalone retrospective or prospective NHS. Optimizing the benefits of the hybrid design as external comparators for SATs requires that investigators first evaluate the suitability of this design, including the type of hybrid study applicable to or feasible for the target disease, and carefully consider specific design and operational elements from study conceptualization to analysis.

This paper supports adoption of this important and at times under-used study design by detailing the methodological, analytic, and regulatory considerations for three hybrid external comparator designs. While not exhaustive, the considerations described here provide a practical framework for investigators as they consider hybrid designs and discuss best practices for handling potential bias in these studies as well as ways to improve methodological rigor. For instance, there is a need to establish and standardize acceptable criteria and assumptions for a head-to-head combination of retrospective and prospective data in the “same patient” designs or a combination of patients who have either retrospective or prospective data in the mixed patient design. Data tokenization is a potential approach to improving data quality and completeness; however, widespread adoption of this approach is currently limited by geographic, ethical, and operational challenges. While not the focus of this manuscript, methods to simplify data tokenization processes while protecting patient information may facilitate broader adoption of this process and improve the quality of hybrid external comparator data.

Although case studies of hybrid external comparator designs are rare in the literature and guidance on their use to support regulatory approval is currently not included in publicly available regulatory documents, we anticipate that hybrid NHS will be increasingly leveraged as external comparators to support drug development in rare diseases. Investigators who intend to adopt this design are encouraged to engage early with the regulatory authorities. As the adoption of this design increases, there is a need for more guidance from regulators on scenarios in which hybrid designs should be considered acceptable as external comparators, as well as best practices for the definition of study cohorts, time zero, and analytical methods to mitigate potential biases.
